# Gender as a moderator of the association between academic self-efficacy and academic performance among nursing students in Saudi Arabia

**DOI:** 10.1371/journal.pone.0359418

**Published:** 2026-09-25

**Authors:** Haidee Pacheco, Richard Dennis Dayrit, Larry Terence Cornejo, Shaimaa Nageeb, Asia Saad Alrashidi, Maha Sanat Alrashidi, Nojoud Abdullah Alrashidi, Jordan Llego

**Affiliations:** 1 Medical Surgical Nursing Department, College of Nursing, University of Ha’il, Ha’il, Saudi Arabia; 2 Nursing Administration Department, College of Nursing, University of Ha’il, Ha’il, Saudi Arabia; 3 Psychiatric and Mental Health Nursing Department, College of Nursing, University of Ha’il, Ha’il, Saudi Arabia; 4 Psychiatric and Mental Health Nursing, Faculty of Nursing, Zagazig University, Zagazig, Egypt; 5 Maternity and Child Health Nursing Department, College of Nursing, University of Ha’il, Ha’il, Saudi Arabia; 6 Nursing Administration and Education Department, College of Nursing and Midwifery, University of Luzon, Dagupan City, Philippines; 7 Office of the Assistant Vice President for Research, Quality Assurance, Accreditation and Curriculum Development, HCT Academy, Pasig City, Philippines; Ege University Faculty of Medicine, TÜRKIYE

## Abstract

**Background:**

Academic performance in nursing education is influenced by multiple psychosocial factors, including academic self-efficacy, defined as students’ confidence in their ability to complete academic learning tasks successfully, which is consistently relevant to student achievement and adaptation. Gender disparities in academic self-efficacy and academic achievement have been observed across educational settings; however, limited research has examined whether gender moderates the relationship between academic self-efficacy and academic performance among nursing students in Saudi Arabia, particularly during curriculum transition.

**Aim:**

This study aimed to explore the relationship between academic self-efficacy and academic performance among nursing students at the University of Hail, Saudi Arabia, and investigate whether gender moderates this relationship.

**Methodology:**

A quantitative cross-sectional comparative-correlational design was used among 441 fourth-year nursing students from two cohorts under a revised curriculum. Using total enumeration, all 458 eligible students were invited; 441 completed the survey. Academic self-efficacy was measured using the eight-item Self-Efficacy for Learning and Performance subscale of the Motivated Strategies for Learning Questionnaire (MSLQ), whereas academic performance was based on registrar-obtained cumulative Grade Point Average (GPA). Because self-efficacy was measured in the fourth year and GPA incorporated earlier academic performance, analyses were interpreted as associations rather than temporal predictions. Data were analyzed using descriptive statistics, independent-samples t tests with Cohen’s d, Pearson correlation, and hierarchical multiple regression with a mean-centered interaction term.

**Results:**

The self-efficacy subscale demonstrated good internal consistency (Cronbach’s alpha = .870). Students reported high academic self-efficacy (M = 5.33, SD = 0.89) and Very Good academic performance (GPA M = 3.20, SD = 0.38). Female students had significantly higher GPA than male students, t(439) = −2.818, p = .005, d = −0.27, although academic self-efficacy did not differ significantly by gender, t(439) = 1.200, p = .231. The first cohort had significantly higher GPA than the second cohort, t(439) = 6.310, p < .001, d = 0.60, whereas the cohort difference in academic self-efficacy was not significant. Academic self-efficacy had a weak positive association with GPA, r(439) =.103, p = .030. Gender did not significantly moderate this association, delta R-squared = .004, F change(1, 437) = 1.641, p = .201.

**Conclusion:**

Nursing students demonstrated high academic self-efficacy and Very Good academic performance. Fourth-year academic self-efficacy was positively but weakly associated with cumulative GPA, and this association did not differ significantly by gender. Because the variables were measured cross-sectionally and cumulative GPA included earlier academic performance, the findings do not establish that current self-efficacy predicted past achievement. Nursing education programs should therefore consider broader academic, curricular, psychosocial, and contextual factors when designing student support strategies.

## Introduction

Academic performance in nursing education is consequential for students’ professional development and for preparing a competent nursing workforce. It reflects interacting academic, clinical, motivational, and contextual influences rather than a single determinant [1–3]. Among these influences, academic self-efficacy refers specifically to students’ confidence in learning course content, completing academic tasks, and meeting performance demands. It is conceptually distinct from general confidence and is the construct assessed by the Self-Efficacy for Learning and Performance subscale of the Motivated Strategies for Learning Questionnaire (MSLQ).

Bandura’s Social Cognitive Theory describes efficacy beliefs as task-specific judgments shaped by mastery experiences, vicarious learning, social persuasion, and affective or physiological states; these beliefs may influence effort, persistence, and self-regulation [4–6]. In nursing and healthcare education, academic self-efficacy has been associated with achievement, engagement, learning strategies, academic satisfaction, and burnout [7–11]. The strength of these associations nevertheless varies across samples and educational contexts, making direct examination necessary rather than assuming that self-efficacy is a strong determinant of GPA.

Saudi nursing students encounter concurrent theoretical, laboratory, and clinical demands, while curriculum transitions can add unfamiliar course structures, assessment expectations, and adjustment pressures. Recent Saudi studies have examined academic and clinical stress, learning environments, academic reform, self-efficacy, and clinical performance anxiety [12–17]. However, evidence remains limited on the association between academic self-efficacy and cumulative GPA among nursing students completing a revised curriculum.

Gender may also shape academic experiences through socialization, institutional expectations, stereotypes, role models, and access to learning opportunities. Gender differences have been reported in self-efficacy, motivation, academic self-concept, and achievement, but their direction and magnitude are not consistent across disciplines and sociocultural settings [18–21]. It is therefore important to test whether gender changes the strength of the self-efficacy-GPA association in the Saudi nursing context rather than treating gender as a uniformly explanatory factor.

This study examined academic self-efficacy, cumulative GPA, gender and cohort differences, and the moderating role of gender among fourth-year nursing students at the University of Hail. Descriptive characteristics were reported to contextualize the sample but were not framed as separate research questions. The study addressed five questions: (1) What are the levels of academic self-efficacy and academic performance among the respondents? (2) Do academic self-efficacy and academic performance differ by gender? (3) Do academic self-efficacy and academic performance differ by cohort? (4) Is academic self-efficacy associated with cumulative GPA? and (5) Does gender moderate the association between academic self-efficacy and cumulative GPA?

## Methodology

### Design

A quantitative cross-sectional comparative-correlational design was used. The comparative component examined differences in academic self-efficacy and academic performance according to gender and cohort. The correlational component examined the relationship between academic self-efficacy and academic performance. Hierarchical multiple regression was used to determine whether gender moderated the relationship between academic self-efficacy and academic performance.

### Setting

The study was conducted at the College of Nursing, University of Hail, Saudi Arabia.

### Population

The study population consisted of fourth-year nursing students from the first two cohorts enrolled under the revised curriculum, with cumulative GPA records available from the university registrar.

### Inclusion criteria

Participants were eligible if they were fourth-year nursing students enrolled at the University of Hail, belonged to either of the first two cohorts under the revised curriculum, and had the institutional demographic and cumulative GPA records required for data linkage.

### Exclusion criteria

Students were excluded if they were not in the fourth year, were not members of the two eligible cohorts as verified through their university ID numbers, declined participation, were unavailable during the data collection period, did not complete the academic self-efficacy questionnaire, or had unavailable registrar GPA data.

### Sample and sampling technique

A total-enumeration approach was used: all 458 eligible students from the two cohorts were invited to participate. Of these, 441 completed and submitted the online questionnaire; all 441 were included in the analysis, yielding a participation rate of 96.3%. The term cohort refers to the first and second groups of students enrolled under the revised curriculum.

### Research instrument

A two-part research instrument was employed. The first section gathered the student ID number, gender category, and year level needed for eligibility verification and linkage. Gender was recorded using the two categories available in the institutional enrollment data for these cohorts (male and female); the source record did not provide a non-binary or self-described option. This administrative constraint determined the categories available for analysis and should not be interpreted as asserting that gender is inherently binary.

The second section assessed academic self-efficacy using the Self-Efficacy for Learning and Performance subscale of the Motivated Strategies for Learning Questionnaire (MSLQ) developed by Pintrich et al. [22]. The full MSLQ contains 81 self-report items across motivation and learning-strategy domains; the selected subscale contains eight items rated from 1 (not at all true of me) to 7 (very true of me). The subscale score was calculated as the mean of the eight responses. For descriptive interpretation, the theoretical 1–7 continuum was divided into three equal two-point intervals: Low (1.00–3.00), Moderate (>3.00–5.00), and High (>5.00–7.00). These boundaries were derived from the full response range rather than from the observed sample distribution, and continuous scores were retained for all inferential analyses. The original MSLQ documentation reported a Cronbach’s alpha of.93 for this subscale.

Academic performance was measured using cumulative Grade Point Average (GPA) obtained from the university registrar. GPA incorporated achievement accrued before and through the fourth-year data-collection period and was treated as a continuous variable for inferential analyses. Because academic self-efficacy was measured only in the fourth year, it could not temporally predict GPA earned in Years 1−3; the analysis therefore estimates a cross-sectional association, and prior academic performance may also have shaped current efficacy beliefs. GPA categories were retained solely for descriptive profiling using the University of Hail grading scale: Exceptional, 4.00 and above; Excellent, 3.75–3.99; Superior, 3.50–3.74; Very Good, 3.00–3.49; Above Average, 2.50–2.99; Good, 2.00–2.49; High Pass, 1.50–1.99; and Pass, 1.00–1.49.

Although the target participants were fourth-year students, the year level was included in the demographic section for verification. Student ID numbers were collected solely to verify cohort membership and match responses with registrar-provided GPA data. Responses from students whose ID numbers did not correspond to the eligible cohorts were excluded from the final analysis.

### Validity and reliability of the instrument

Internal consistency was examined in a pilot sample of 30 students from the same college; the pilot participants were not included in the main analysis. The eight-item Self-Efficacy for Learning and Performance subscale demonstrated good internal consistency, with Cronbach’s alpha = .870. Corrected item-total correlations ranged from.564 to.707, and deletion of any item did not improve the overall alpha; therefore, all eight items were retained.

Internal consistency was treated as evidence of reliability, not as evidence of full validity. The Self-Efficacy for Learning and Performance subscale was used because it is an established academic self-efficacy measure and its content directly corresponds to students’ confidence in understanding course materials, mastering academic skills, completing assignments and examinations, and performing well in class. However, a full local psychometric validation of the subscale among Saudi nursing students, including expert content validity assessment, face validity testing, factor structure evaluation, convergent validity, criterion-related validity, and measurement invariance across gender, was not conducted as part of this study.

### Data gathering procedure

Data collection was conducted in two periods. Data for the first cohort were collected during the second semester of academic year 2022–2023, from January to March 2023. Data for the second cohort were collected during the second semester of academic year 2023–2024, from January to March 2024. The survey was administered online using Google Forms and required approximately 5–10 minutes to complete. The link was disseminated only to eligible fourth-year nursing students from the two revised-curriculum cohorts through official class communication channels, including cohort WhatsApp groups. Class leaders assisted in forwarding the survey link, and coordination was made with course faculty to ensure that the invitation reached the intended student groups.

Students’ identification numbers were used only to verify cohort eligibility and link survey responses to cumulative GPA obtained from the university registrar. Because student ID numbers were used for linkage, responses were treated as confidential rather than fully anonymous during data matching. After GPA linkage and data verification, students were assigned unique codes, and the working dataset used for analysis was de-identified and contained no student names or university ID numbers. Access to identifiable information was limited to authorized members of the research team.

### Ethical consideration

The study was approved by the University of Hail Institutional Review Board (IRB) and was designated as Project No. H-2022–353. The purpose of the study and its voluntary nature were disclosed to all potential participants. They were assured of confidentiality throughout the research process and were permitted to disengage from the study at any time without incurring any academic repercussions. Informed consent was obtained by providing it in the online form seen before they start to complete the online questionnaire.

### Statistical analysis

Data were analyzed using IBM SPSS Statistics. Frequencies and percentages described gender, cohort, and GPA category, while means and standard deviations summarized academic self-efficacy and continuous GPA. Independent-samples t tests compared academic self-efficacy and GPA by gender and cohort; homogeneity of variance was examined using Levene’s test before the equal-variance estimates were interpreted. Cohen’s d was calculated for each t-test comparison and interpreted by absolute magnitude as negligible (<0.20), small (0.20–0.49), medium (0.50–0.79), or large (>=0.80). Pearson product-moment correlation examined the association between academic self-efficacy and GPA. Hierarchical multiple regression tested whether gender moderated this association. Linearity was examined using scatterplots, standardized residual-versus-predicted plots, and a joint test of quadratic terms. Homoscedasticity was evaluated using residual plots and the Breusch-Pagan and White tests, while multicollinearity was assessed using tolerance and variance inflation factors (VIFs). Academic self-efficacy was mean centered before the interaction term was created. Model 1 included centered academic self-efficacy and gender; Model 2 added the centered academic self-efficacy x gender interaction. Gender was coded 0 = male and 1 = female solely to represent the two categories available in the institutional data. Because self-efficacy was measured in the fourth year and GPA was cumulative, regression coefficients were interpreted as cross-sectional associations, not evidence that current self-efficacy predicted earlier achievement. Statistical significance was set at p < .05 with 95% confidence intervals.

## Results

### Demographic and academic profile of respondents

A total of 441 fourth-year nursing students were included in the analysis. Female students comprised 54.4% of the sample, while male students comprised 45.6%. More than half of the respondents (55.1%) belonged to the second cohort, while 44.9% belonged to the first cohort. In terms of academic performance category, the largest proportion of students was classified as Very Good (45.6%), followed by Above Average (26.5%) and Superior (17.2%). Only a small proportion of students were classified as Exceptional (0.7%), Good (3.2%), or High Pass (0.2%) (Table 1).

**Table 1:** Demographic and academic profile of the nursing student respondents (n = 441).

Variable	Category	Frequency	Percentage
Gender	Male	201	45.6
	Female	240	54.4
Cohort	First cohort	198	44.9
	Second cohort	243	55.1
GPA category	Exceptional	3	0.7
	Excellent	29	6.6
	Superior	76	17.2
	Very Good	201	45.6
	Above Average	117	26.5
	Good	14	3.2
	High Pass	1	0.2

*Note.* Percentages are based on valid cases. GPA category was used for descriptive profiling only; continuous GPA was used for inferential analyses.

### Academic self-efficacy and academic performance

Students demonstrated high academic self-efficacy, with a mean score of 5.33 (SD = 0.89). The mean continuous GPA was 3.20 (SD = 0.38), which corresponds to Very Good academic performance. When analyzed by gender, both male and female students exhibited high academic self-efficacy and Very Good academic performance. Male students reported a slightly higher mean academic self-efficacy score, while female students achieved a higher mean GPA. Analysis by cohort indicated that both groups demonstrated high academic self-efficacy and Very Good academic performance. The second cohort reported a slightly higher mean academic self-efficacy score, whereas the first cohort achieved a higher mean GPA (Table 2).

**Table 2:** Academic self-efficacy and academic performance overall and according to gender and cohort.

Group	n	Academic Self-Efficacy M (SD)	Interpretation	GPA M (SD)	Interpretation
Overall	441	5.33 (0.89)	High	3.20 (0.38)	Very Good
Male	201	5.39 (0.93)	High	3.14 (0.35)	Very Good
Female	240	5.29 (0.86)	High	3.25 (0.40)	Very Good
First cohort	198	5.29 (0.90)	High	3.32 (0.34)	Very Good
Second cohort	243	5.37 (0.89)	High	3.10 (0.38)	Very Good

*Note.* Academic self-efficacy was measured using the Self-Efficacy for Learning and Performance subscale. GPA was treated as a continuous variable for inferential analyses.

### Differences by gender and cohort

Levene’s tests were nonsignificant for GPA by gender, F(1, 439) = 2.791, p = .095; academic self-efficacy by gender, F(1, 439) = 0.847, p = .358; GPA by cohort, F(1, 439) = 1.621, p = .204; and academic self-efficacy by cohort, F(1, 439) = 0.295, p = .588. Thus, the equal-variance t-test estimates were appropriate. Female students had significantly higher GPA than male students, t(439) = −2.818, p = .005, d = −0.27, a small effect. Academic self-efficacy did not differ significantly by gender, t(439) = 1.200, p = .231, d = 0.11, a negligible effect. The first cohort had significantly higher GPA than the second cohort, t(439) = 6.310, p < .001, d = 0.60, a medium effect, whereas the cohort difference in academic self-efficacy was not significant, t(439) = −0.900, p = .369, d = −0.09, a negligible effect. Table 3 summarizes the inferential results.

**Table 3:** Inferential tests for academic self-efficacy and academic performance.

Analysis	Variable	Test Statistic	p value	Interpretation	Cohen’s d	Magnitude
Gender difference	GPA	t(439) = −2.818	.005	Significant; female students had higher GPA	−0.27	Small
Gender difference	Academic self-efficacy	t(439) = 1.200	.231	Not significant	0.11	Negligible
Cohort difference	GPA	t(439) = 6.310	<.001	Significant; first cohort had higher GPA	0.60	Medium
Cohort difference	Academic self-efficacy	t(439) = −0.900	.369	Not significant	−0.09	Negligible
Correlation	Academic self-efficacy and GPA	r(439) =.103	.030	Significant weak positive relationship	NA	NA

Note. Independent-samples t tests were used for gender and cohort comparisons, and Pearson product-moment correlation examined the association between academic self-efficacy and cumulative GPA. Cohen’s d was calculated as t multiplied by the square root of (1/n1 + 1/n2); signs follow the first-minus-second group order shown in Table 2. Absolute d values were interpreted as negligible (<0.20), small (0.20–0.49), medium (0.50–0.79), or large (>=0.80). NA = not applicable.

### Relationship between academic self-efficacy and academic performance

Pearson correlation analysis showed a weak but statistically significant positive relationship between academic self-efficacy and continuous GPA, r(439) =.103, p = .030. This indicates that students with higher academic self-efficacy tended to have slightly higher GPAs, although the relationship was small (Table 3).

### Moderating role of gender

**Fig 1:**
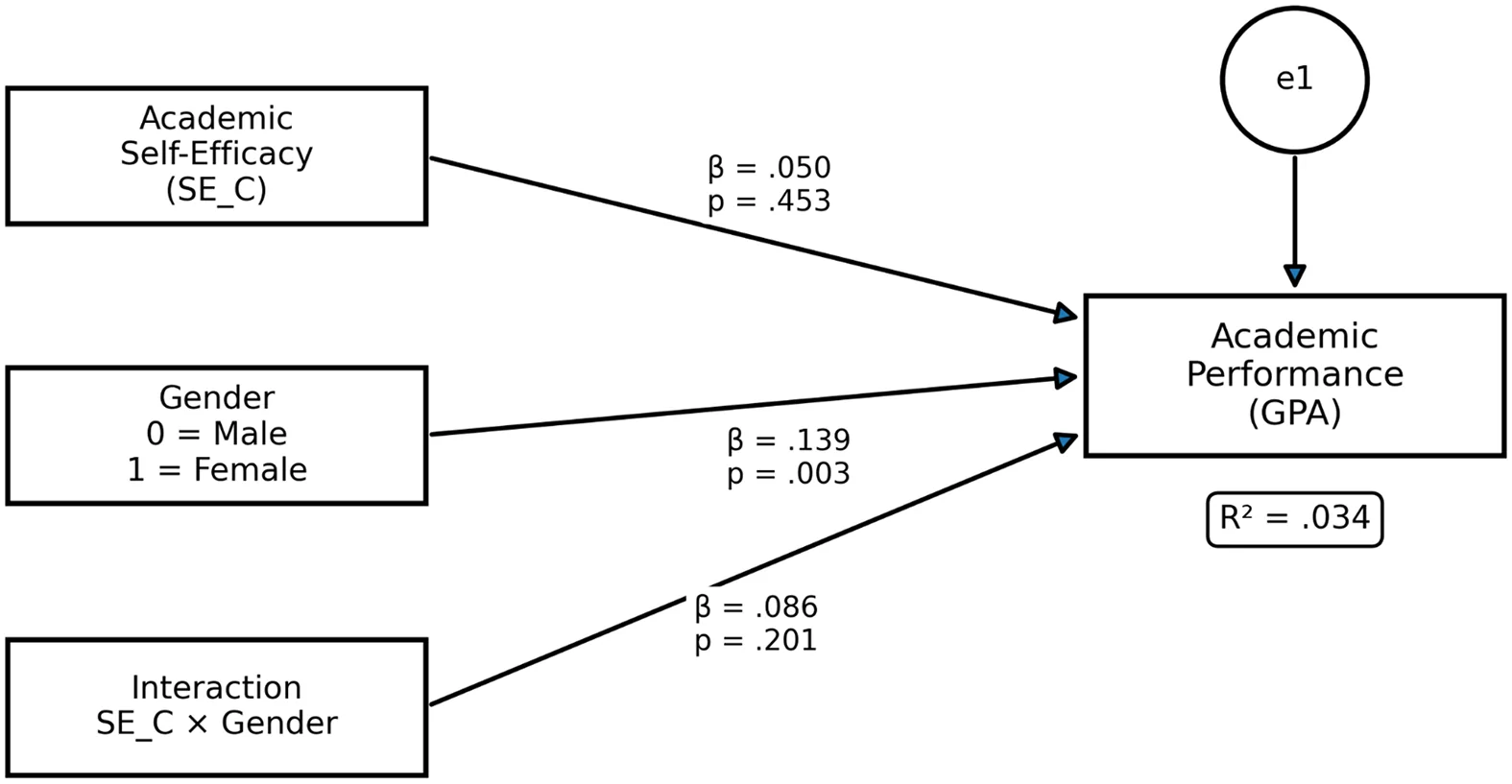
Moderation model for the association between academic self-efficacy and academic performance. Note. SE_C = mean-centered academic self-efficacy; GPA = Grade Point Average; beta = standardized regression coefficient; p = probability value; R-squared = explained variance in academic performance. Gender was coded as 0 = male and 1 = female to represent the two categories available in the institutional data. Arrows represent regression paths and do not establish temporal or causal effects. The interaction term was not statistically significant. e1 represents the residual error term.

Hierarchical multiple regression was conducted to determine whether gender moderated the association between academic self-efficacy and GPA. The residual-versus-predicted plot did not show a material curve or funnel pattern. A joint test of quadratic self-efficacy terms was nonsignificant, F(2, 435) = 1.360, p = .258, and the Breusch-Pagan test, chi-square(3) = 4.125, p = .248, and White test, chi-square(5) = 4.692, p = .455, did not indicate heteroscedasticity. VIFs ranged from 1.00 to 2.02 (tolerance = .49–1.00), indicating no problematic multicollinearity. In Model 1, academic self-efficacy and gender were jointly associated with GPA, F(2, 438) = 6.789, p = .001, R-squared = .030. Adding the academic self-efficacy x gender interaction in Model 2 produced a small, non-significant increase in explained variance, delta R-squared = .004, F change(1, 437) = 1.641, p = .201. The unrounded increment was approximately.0036, which rounds to.004; F change was calculated from the unrounded model estimates. Gender therefore did not significantly moderate the self-efficacy-GPA association (Table 4; Fig 1).

**Table 4:** Hierarchical regression analysis testing the moderating role of gender.

Model	Model terms	R	R²	Adjusted R²	ΔR²	F Change	p value
Model 1	ASE; Gender	.173	.030	.026	.030	6.789	.001
Model 2	ASE; Gender; ASE × Gender	.184	.034	.027	.004	1.641	.201

Note. Dependent variable = continuous GPA. Gender was coded 0 = male and 1 = female to represent the two institutional categories available for analysis. ASE = academic self-efficacy. Academic self-efficacy was mean-centered before the interaction term was created. The unrounded R-squared increment was approximately.0036, which rounds to the displayed.004; F change was calculated from the unrounded model estimates. Regression terms represent cross-sectional associations and do not establish temporal prediction.

## Discussion

The present study examined academic self-efficacy, academic performance, gender differences, cohort differences, and the moderating effect of gender among fourth-year nursing students at the University of Hail. The findings revealed that students exhibited high academic self-efficacy and attained very good academic performance. Female students achieved significantly higher GPAs compared to male students. Although male students reported a slightly higher mean academic self-efficacy score than female students, this difference was not statistically significant. The first cohort obtained a significantly higher GPA than the second cohort, while the second cohort demonstrated a marginally higher mean academic self-efficacy score than the first cohort; however, this difference was also not statistically significant. Academic self-efficacy was weakly but significantly and positively correlated with academic performance. Furthermore, gender did not significantly moderate the relationship between academic self-efficacy and academic performance.

A total of 441 nursing students participated, with females comprising 54.4% of the sample. This distribution was more balanced than the 75.7% female composition reported in a primary study of 338 nursing students at the same University of Hail [23], underscoring that gender composition can vary by cohort and sampling frame and should not be generalized as a global trend. Most participants in the present study attained a Very Good cumulative GPA. This pattern differs from the higher GPA profile reported by Oliveira Silva [24] and the varied GPA distribution observed by Yeo et al. [25].

The observation of high academic self-efficacy among fourth-year nursing students is theoretically plausible, given their accumulated academic familiarity, mastery experiences, clinical exposure, and repeated feedback from faculty and peers. Bandura’s social cognitive theory identifies mastery experience and social persuasion as key sources of efficacy beliefs [6]. This interpretation is supported by Ersoy and Ayaz-Alkaya [26], who found that fourth-year nursing students exhibited higher academic self-efficacy than students in lower year levels, and that participation in clinical practice, care-plan preparation, and case discussions was associated with stronger academic self-efficacy and readiness for professional practice. The present finding is also consistent with recent studies reporting favorable or high academic self-efficacy among nursing students in both academic and clinical learning contexts [27–31]. However, high academic self-efficacy should not be assumed to be universal among nursing students, as academic burnout, psychological distress, perceived stressors, and poor-quality learning experiences may diminish students’ confidence [16,30,31]. Therefore, the high self-efficacy observed in this study may reflect respondents’ senior-year status, accumulated academic and clinical exposure, institutional support, and adaptation to academic demands.

Female students attained significantly higher GPAs than male students. This finding is consistent with research indicating that female students may achieve higher grade-based academic outcomes across diverse educational contexts. For instance, Al-Dweik et al. [32] reported higher face-to-face and online GPAs among female university students in Jordan, and Tsaousis and Alghamdi [20] found that females scored higher than males in the GPA domain among Saudi high school graduates applying for higher education. Although males may have higher university entrance scores, females frequently achieve better cumulative academic results [33]. Conversely, evidence from Ethiopia indicated that male students outperformed female students in CGPA and national exit examination results [34], and Nasir et al. [35] reported higher academic performance scores among male health sciences students in Saudi Arabia.

The higher GPA observed among female students may be partially explained by classroom engagement and academic responsibility within the College of Nursing at the University of Hail. Although variables such as attendance, diligence, study habits, and task completion were not directly measured, faculty observations indicate that female students are generally more consistent in attending lectures and fulfilling academic requirements than male students. Similar informal feedback has been received from male faculty members, despite the gender-segregated learning environment. These observations are pertinent because GPA is cumulative and may reflect sustained academic behaviors, including regular attendance, preparation, time management, effort regulation, and persistence. Recent studies corroborate this interpretation. Liu et al. [36] reported gender differences in self-regulated learning, including task strategies, time management, self-evaluation, and help-seeking, while Xu et al. [37] found that self-regulated learning strategies were associated with GPA-related academic performance.

Within the context of nursing education, this finding should also be considered in relation to the gendered nature of the profession. Nursing has historically been associated with women, and male nursing students may encounter challenges related to gender norms, stereotypes, role expectations, professional identity formation, or a lack of male role models in academic and clinical settings [38,39]. Although these factors were not directly measured in the present study, they may contribute to differences in academic performance patterns between male and female nursing students across various contexts.

Accordingly, the higher GPA among female students in this study should be interpreted as a context-specific finding rather than as evidence of inherent gender-based academic superiority. This result may reflect differences in academic engagement, learning strategies, assessment-related behaviors, motivation, or adaptation to nursing program expectations. However, these explanatory variables were not measured and should be directly examined in future research [40].

Although male students reported a slightly higher mean academic self-efficacy score than female students, this difference was not statistically significant. A similar gender pattern was observed by Ozsaker et al. [31], who found that male nursing students had significantly higher academic self-efficacy scores than female nursing students. Likewise, Nordhus et al. [27] and Purwandari et al. [28] documented higher levels of self-efficacy among nursing students in Norway and Indonesia, respectively. These findings suggest that male nursing students may sometimes report greater confidence in their academic ability and learning performance. The absence of a significant gender difference in academic self-efficacy in the present study is consistent with Türker and Bulut [41], who also reported no significant gender difference among nursing students. Therefore, the slightly higher self-efficacy among male students in this study should be interpreted as a descriptive pattern rather than a confirmed gender-based difference. The lack of a significant gender difference indicates that male and female students had comparable confidence in their academic ability. This may be partially explained by their shared exposure to the same curriculum, course requirements, examinations, clinical training expectations, academic standards, and faculty feedback. According to social cognitive theory, academic self-efficacy develops through mastery experiences, vicarious experiences, verbal persuasion, and physiological or emotional states; thus, students exposed to similar academic and clinical learning conditions may develop broadly comparable efficacy beliefs [6]. Longitudinal evidence in nursing education also suggests that academic self-efficacy may change over the course of training and may be shaped by students’ educational experiences [42]. Recent research further indicates that gender differences in self-efficacy are not uniform across educational contexts and may vary according to discipline, learning environment, sociocultural expectations, and measurement approach [18,21,43].

Previous studies also indicate that gender differences in self-efficacy are inconsistent and may vary across academic fields, cultural contexts, gender stereotypes, and learning environments [18,21,44].

A significant cohort difference in GPA was identified, with the first cohort achieving a higher mean GPA than the second cohort. Since both cohorts were enrolled under the revised curriculum, this difference may reflect variation in curriculum implementation periods rather than differences in curricular structure. Curriculum implementation in nursing education is a developmental process that may involve faculty adjustment, changes in teaching strategies, institutional support, learner engagement, and adaptation to new academic expectations [15,45]. However, the present study did not directly assess cohort-level factors such as academic preparedness, teaching and assessment conditions, academic monitoring, student adjustment, psychological distress, or stress during curriculum implementation. Consequently, the reason for the higher GPA in the first cohort cannot be determined from the available data. Recent literature in nursing education suggests that academic reform, academic advising, psychological distress, academic burnout, and the quality of learning experiences may influence students’ academic adjustment and performance [15,16].

Although both cohorts demonstrated high academic self-efficacy, the second cohort reported a slightly higher mean academic self-efficacy score than the first cohort; however, this difference was not statistically significant. This finding indicates that both cohorts had broadly comparable confidence in their academic capabilities. One possible contextual explanation is that the second cohort experienced the revised curriculum after the institution and faculty had already implemented it with the first cohort, potentially clarifying course delivery, assessment expectations, advising processes, and academic support mechanisms. The College of Nursing provides academic advising mechanisms to monitor student progress, support academically weak students, and encourage high-performing students. However, individual students’ exposure to academic advising, use of remedial support, or receipt of specific feedback was not measured in this study. Therefore, the role of academic advising should be considered a plausible contextual explanation rather than a demonstrated cause. This interpretation aligns with recent evidence emphasizing academic advising and institutional support during academic reform among Saudi nursing students [15].

The weak positive association indicates that students reporting greater confidence in understanding course material and completing academic tasks tended to have slightly higher cumulative GPA. Two measurement features temper this finding. First, self-efficacy was assessed in the fourth year, whereas cumulative GPA incorporated substantial achievement from Years 1–3; consequently, current self-efficacy cannot explain earlier grades, and prior success or difficulty may itself have shaped present efficacy beliefs. Second, the high mean self-efficacy score (M = 5.33 on a 1–7 scale) suggests clustering toward the upper part of the scale. Such range restriction can attenuate correlations and interaction effects by reducing between-student variability. The observed r = .103 should therefore be interpreted as a small contemporaneous association within this relatively high-efficacy sample, not as evidence that fourth-year self-efficacy predicted past GPA. This cautious interpretation is consistent with Atak et al. [46], who also reported a weak positive association between academic self-efficacy and academic average among nursing students.

In addition to confirming the association between academic self-efficacy and academic outcomes, previous studies have identified possible mechanisms underlying this relationship. Hayat et al. [9] found that academic self-efficacy was related to improved academic performance through metacognitive learning strategies and learning-related emotions. Bulfone et al. [8] reported that burnout mediated the relationship between self-efficacy and academic success among nursing students. In the current context, academic advising, early monitoring of weak performance and absenteeism, remedial learning opportunities, and formative feedback may support students’ confidence and academic adjustment. However, as the present study did not directly measure students’ use of academic advising services, remedial classes, feedback mechanisms, or burnout, these institutional practices should be considered possible supportive explanations rather than confirmed causal mechanisms.

The relationship between self-efficacy and academic performance is not uniformly weak. For example, Zhang [47] reported a very strong positive correlation between self-efficacy and academic performance among Chinese medical undergraduates. These findings indicate that the strength of the self-efficacy–performance relationship may vary across disciplines, samples, learning contexts, measurement approaches, and assessment methods. Therefore, while academic self-efficacy is relevant to academic performance, the weak correlation observed in the present study suggests that confidence alone does not fully account for GPA. Academic success in nursing and healthcare education is multidimensional and may also be influenced by engagement, self-regulation, course demands, study habits, grading practices, academic support, and other contextual factors [10,48].

The moderation analysis indicated that gender did not significantly alter the relationship between academic self-efficacy and academic performance. In practical terms, the weak positive association between academic self-efficacy and academic performance was similar for both male and female students. This result contrasts with broader educational literature suggesting that gender may sometimes moderate relationships involving academic self-beliefs, self-efficacy, motivation, and achievement. For example, Wang and Yu [21] concluded that gender may moderate the effects of academic self-concept on achievement, motivation, performance, and self-efficacy. Similarly, Zhao et al. [49] found that gender had a moderating influence in a model involving student self-management, self-efficacy, and academic achievement, and Khalid and Rahman [50] observed gender moderation in the relationship between academic self-efficacy and achievement among Malaysian students. However, gender moderation is not consistently observed across all educational contexts, and gender-related effects may vary across academic domains, student populations, measurement approaches, and sociocultural settings [21,43]. In the present study, male and female students followed the same curriculum, course requirements, assessment standards, and clinical expectations. These shared academic conditions may have contributed to the similar relationship between academic self-efficacy and academic performance across gender groups, as self-efficacy is shaped by mastery experiences, feedback, and repeated academic and clinical learning experiences [6,42]. Therefore, gender did not condition this specific relationship in the present sample.

### Limitation

Several limitations should be considered. First, academic self-efficacy was measured in the fourth year, while cumulative GPA included performance accrued in earlier years. The temporal ordering required for prediction was therefore absent: the study estimates an association, and reciprocal influence from prior achievement to current self-efficacy is plausible. Second, the high self-efficacy mean and concentration of respondents in the High category may have restricted score variability and attenuated the observed correlation and interaction. Third, self-efficacy was self-reported and may be affected by social desirability, response style, or current emotional state. Fourth, only the Self-Efficacy for Learning and Performance subscale of the MSLQ was used, and a full local psychometric validation, including measurement invariance across gender, was not conducted. Fifth, the single-institution setting limits generalizability. Sixth, gender analysis was restricted to the male and female categories available in the institutional enrollment record; the study did not collect a non-binary or self-described gender option and cannot represent more diverse identities. Seventh, registrar-derived GPA may still reflect differences in course difficulty, assessment structure, and grading practices. Finally, online data collection may have introduced selection or response bias, and unmeasured cohort-level conditions limit explanation of the observed cohort difference.

## Conclusion

This study found that fourth-year nursing students at the University of Hail demonstrated high academic self-efficacy and Very Good academic performance. Academic self-efficacy was weakly but significantly and positively related to academic performance. Female students had significantly higher GPA than male students, while male and female students did not differ significantly in academic self-efficacy. The first cohort had significantly higher GPA than the second cohort, but cohort differences in academic self-efficacy were not significant. Gender did not significantly moderate the relationship between academic self-efficacy and academic performance. These findings suggest that academic self-efficacy is relevant to academic performance but explains only a small proportion of GPA variation. Nursing education programs should therefore address academic performance through comprehensive strategies that include academic self-efficacy, learning support, assessment preparation, curricular monitoring, advising, and psychosocial support.

### Recommendations

Nursing education programs should continue to strengthen academic self-efficacy through structured learning experiences that promote mastery, constructive feedback, guided reflection, simulation-based learning, academic skills training, enhanced academic advising, and timely remediation. Recent intervention and correlational evidence support the value of educational strategies that build self-efficacy and related competencies among nursing students, including talent management interventions, academic skills training, active teaching methods, mentoring, and structured educational support [51–54]. These strategies should be integrated with monitoring of actual academic performance so that students who appear confident but underperforming can still receive targeted support.

Future studies should use longitudinal designs to examine how academic self-efficacy and GPA influence each other over time. Multi-institutional studies are also recommended to improve generalizability across Saudi nursing programs. Future research should include additional predictors such as learning strategies, academic stress, language proficiency, motivation, teaching quality, clinical learning environment, socioeconomic factors, perceived social support, academic advising, and student engagement. Qualitative studies may also help explain how nursing students experience confidence, gender expectations, cohort differences, and academic performance within the Saudi educational context.

## Supporting information

S1 Dataset Participant-level data on academic self-efficacy and academic performance. (XLSX)
