# Supplementary material for: Gender as a moderator of the association between academic self-efficacy and academic performance among nursing students in Saudi Arabia
Source: PLoS One. 2026 Sep 25;21(9):e0359418. doi: 10.1371/journal.pone.0359418 (PMC13614655; doi:10.1371/journal.pone.0359418)
Supplement: Supplementary file 1 [file pone.0359418.s002.docx]

Dear Editor and Authors,

Greetings!

Thank you for the opportunity to review this paper. The paper has several methodological and contextual strengths. However, I would like to point few concerns for improvement.

- The academic self-efficacy was measured in **Year 4**, but cumulative GPA includes Years 1–3. Year 4 self-efficacy cannot logically predict or explain past GPA. Please clarify this.
- Please mention of Levene’s test (for t-tests), linearity/homoscedasticity checks, or VIF values for the regression models.
- The gender is coded 0/1, which erases non-binary identities. The limitation mentions this briefly but does not explain why the institutional data only permitted binary coding.
- Please take not that self-efficacy M = 5.33 on a 1–7 scale, with most students categorized as "High." Range restriction artificially attenuates correlation magnitude.
