# Supplementary material for: Gender as a moderator of the association between academic self-efficacy and academic performance among nursing students in Saudi Arabia
Source: PLoS One. 2026 Sep 25;21(9):e0359418. doi: 10.1371/journal.pone.0359418 (PMC13614655; doi:10.1371/journal.pone.0359418)
Supplement: Supplementary file 2 [file pone.0359418.s003.docx]

Dear Authors and Editor,

I am grateful for this invite to review this paper titled “ Gender as a Moderator of the Association Between Academic Self-Efficacy and Academic Performance Among Nursing Students in Saudi Arabia”. This paper is of great interest especially in the context of nursing education. That said, I would like to suggest for the improvement of this paper before publication. Please see below:

1. The introduction is too long. Please make sure that only the elements of the introduction must be present. Also, nine research questions are listed; several (RQ2–RQ5) are purely descriptive and redundant with the profiling questions.i suggest that you have to condense into 4 or 5 focused questions. Note that descriptive profiling does not require separate research questions.
2. Please revisit the ethics statement as it says "All participants provided informed consent." On the part of the paper, it says, “Implied informed consent was obtained."	Use one consistent term throughout. If it was truly "implied" via survey completion, state that in the Ethics Statement too.
3. While it is acceptable, I would still ask the authors to please make justification on the Self-efficacy categorization cutoffs (Low 1.0–2.9, Moderate 3.0–5.0, High 5.1–7.0) are uneven and the Moderate band is 2.0 points wide while High is 1.9.
4. Please add Cohen's d for gender GPA difference and cohort GPA difference as there is no effect sizes (Cohen's d) reported for significant t-tests.
5. There are citation problem that authors need to consider. For instance, page 16	"This gender distribution is consistent with global trends in nursing education (Esht et al., 2023), but contrasts with the more balanced gender ratio observed by Alsadaan et al. (2021)..."	Verify that Alsadaan et al. (2021) actually reports gender ratios of nursing students. The title is "Challenges facing the nursing profession in Saudi Arabia: An integrative review" …if it does not report student gender distributions, replace the citation with a primary source that does.
6. The table reported as ΔR² = .004; F change = 1.641. Would this numbers be true at the same time? Please check.
7. There is a need also to report the Cohen’s d. Please add the Cohen's d values as two new columns in Table 3.

Thank you
