# Supplementary material for: Gender as a moderator of the association between academic self-efficacy and academic performance among nursing students in Saudi Arabia
Source: PLoS One. 2026 Sep 25;21(9):e0359418. doi: 10.1371/journal.pone.0359418 (PMC13614655; doi:10.1371/journal.pone.0359418)
Supplement: Supplementary file 3 [file pone.0359418.s005.docx]

Dear Editor and Reviewers,

Thank you for the constructive reviews. We have addressed each comment in the revised manuscript. The comments are reproduced verbatim below; all changes are highlighted in yellow. Page references correspond to the revised highlighted manuscript.

| **Numbered Reviewer Comments** | **Responses and Locations of Changes** |
| --- | --- |
| **Reviewer 1 – Comment 1**  “The academic self-efficacy was measured in Year 4, but cumulative GPA includes Years 1–3. Year 4 self-efficacy cannot logically predict or explain past GPA. Please clarify this.” | **Response:** We clarified that fourth-year academic self-efficacy cannot be interpreted as causing or predicting GPA earned during Years 1–3. The regression models are now described as cross-sectional association models, and the Discussion recognizes the plausible reciprocal pathway in which prior academic performance may influence current self-efficacy.  **Location of changes:** Abstract—Methodology and Conclusion (pp. 2–3); Research Instrument (p. 5); Statistical Analysis (p. 7); Discussion (p. 11); and Limitations (pp. 12–13). |
| **Reviewer 1 – Comment 2**  “Please mention of Levene’s test (for t-tests), linearity/homoscedasticity checks, or VIF values for the regression models.” | **Response:** We reanalyzed the raw data and added the exact assumption-check results. Levene's tests were nonsignificant for GPA by gender, F(1, 439) = 2.791, p = .095; academic self-efficacy by gender, F(1, 439) = 0.847, p = .358; GPA by cohort, F(1, 439) = 1.621, p = .204; and academic self-efficacy by cohort, F(1, 439) = 0.295, p = .588. Therefore, the equal-variance t-test estimates were appropriate. For the full regression model, the residual-versus-predicted plot showed no material curve or funnel pattern; the joint test of quadratic terms was nonsignificant, F(2, 435) = 1.360, p = .258; the Breusch-Pagan and White tests were nonsignificant (p = .248 and p = .455, respectively); and VIFs ranged from 1.00 to 2.02 (tolerance = .49-1.00). These results do not indicate material violations of linearity, homoscedasticity, or multicollinearity.  **Location of changes:** Statistical Analysis section; Results—Gender and Cohort Differences; and Results—Moderation Analysis. |
| **Reviewer 1 – Comment 3**  “The gender is coded 0/1, which erases non-binary identities. The limitation mentions this briefly but does not explain why the institutional data only permitted binary coding.” | **Response:** We explained that the institutional enrollment file contained only male and female categories. The 0/1 coding is now identified as an analytic convention for those available categories, not a claim that gender is inherently binary. The limitation acknowledges that students with other gender identities could not be represented or analyzed.  **Location of changes:** Research Instrument (p. 5); Statistical Analysis (p. 7); Figure 1 note (p. 8); Limitations (p. 13); and Table 4 note (p. 21). |
| **Reviewer 1 – Comment 4**  “Please take not that self-efficacy M = 5.33 on a 1–7 scale, with most students categorized as "High." Range restriction artificially attenuates correlation magnitude.” | **Response:** We added an explicit discussion of range restriction. The revised manuscript explains that the high mean and clustering of self-efficacy scores may have reduced variability and thereby attenuated both the observed correlation and the interaction estimate. The small correlation (r = .103) is now interpreted cautiously.  **Location of changes:** Discussion (p. 11) and Limitations (pp. 12–13). |
| **Reviewer 2 – Comment 1**  “The introduction is too long. Please make sure that only the elements of the introduction must be present. Also, nine research questions are listed; several (RQ2–RQ5) are purely descriptive and redundant with the profiling questions.i suggest that you have to condense into 4 or 5 focused questions. Note that descriptive profiling does not require separate research questions.” | **Response:** We shortened the Introduction to retain the study context, research gap, rationale, and purpose. We also reduced the nine research questions to five focused questions. Participant and variable profiling are now treated as descriptive analyses rather than as separate research questions.  **Location of changes:** Condensed Introduction and revised research questions (pp. 3–4). |
| **Reviewer 2 – Comment 2**  “Please revisit the ethics statement as it says "All participants provided informed consent." On the part of the paper, it says, “Implied informed consent was obtained." Use one consistent term throughout. If it was truly "implied" via survey completion, state that in the Ethics Statement too.” | **Response:** We standardized the manuscript to use the term ‘informed consent.’ The revised text explains that participants received the inform consent before answering the online survey.  **Location of changes:** Ethical Consideration (p. 6). |
| **Reviewer 2 – Comment 3**  “While it is acceptable, I would still ask the authors to please make justification on the Self-efficacy categorization cutoffs (Low 1.0–2.9, Moderate 3.0–5.0, High 5.1–7.0) are uneven and the Moderate band is 2.0 points wide while High is 1.9.” | **Response:** We replaced the uneven categories with equal-width theoretical intervals across the 1–7 scale: Low = 1.00–3.00, Moderate = >3.00–5.00, and High = >5.00–7.00. We also clarified that the categories are used only for descriptive presentation; the continuous self-efficacy score is retained for inferential analyses.  **Location of changes:** Research Instrument (p. 5). |
| **Reviewer 2 – Comment 4**  “Please add Cohen's d for gender GPA difference and cohort GPA difference as there is no effect sizes (Cohen's d) reported for significant t-tests.” | **Response:** We calculated and reported Cohen’s d for the significant GPA comparisons. The gender difference was small (d = −0.27), whereas the cohort difference was medium (d = 0.60). The sign follows the group order shown in Table 3.  **Location of changes:** Abstract—Results (p. 3); Results (pp. 7–8); and Table 3 (p. 20). |
| **Reviewer 2 – Comment 5**  “There are citation problem that authors need to consider. For instance, page 16 "This gender distribution is consistent with global trends in nursing education (Esht et al., 2023), but contrasts with the more balanced gender ratio observed by Alsadaan et al. (2021)..." Verify that Alsadaan et al. (2021) actually reports gender ratios of nursing students. The title is "Challenges facing the nursing profession in Saudi Arabia: An integrative review" …if it does not report student gender distributions, replace the citation with a primary source that does.” | **Response:** We removed the disputed comparison and the Esht et al. and Alsadaan et al. citations. We replaced them with a primary Saudi nursing-student study from the same university: Alshammari and Alboliteeh (2022), which reported that 75.7% of 338 respondents were female. The revised discussion does not make the unsupported global comparison.  **Location of changes:** Discussion (pp. 8–9) and References (p. 15). |
| **Reviewer 2 – Comment 6**  “The table reported as ΔR² = .004; F change = 1.641. Would this numbers be true at the same time? Please check.” | **Response:** We checked the internal mathematical consistency of the reported values. The unrounded R² increment is approximately .0036, which rounds to .004; the F-change value of 1.641 was calculated from the unrounded model estimates. We added a note so that the apparent discrepancy caused by rounding is transparent.  **Location of changes:** Abstract—Results (p. 3); Results (p. 8); and Table 4 note (p. 21). |
| **Reviewer 2 – Comment 7**  “There is a need also to report the Cohen’s d. Please add the Cohen's d values as two new columns in Table 3.” | **Response:** We added ‘Cohen’s d’ and ‘Magnitude’ columns to Table 3. The effect sizes are: gender GPA, d = −0.27 (small); gender self-efficacy, d = 0.11 (negligible); cohort GPA, d = 0.60 (medium); and cohort self-efficacy, d = −0.09 (negligible). The table note states the calculation and sign convention.  **Location of changes:** Table 3 and its note (p. 20). |

| **Editorial Comments** | **Responses** |
| --- | --- |
| 1. In the online submission form, you indicated that: The datasets used and/or analyzed during the current study are available from the main author on reasonable request.  All PLOS journals now require all data underlying the findings described in their manuscript to be freely available to other researchers, either 1. In a public repository, 2. Within the manuscript itself, or 3. Uploaded as supplementary information.  This policy applies to all data except where public deposition would breach compliance with the protocol approved by your research ethics board. If your data cannot be made publicly available for ethical or legal reasons (e.g., public availability would compromise patient privacy), please explain your reasons on resubmission and your exemption request will be escalated for approval. | We attached the raw data in our submission as “Supporting Information- RAW DATA SET.” Also, we changed the availability statement. |
| 2. Your ethics statement should only appear in the Methods section of your manuscript. If your ethics statement is written in any section besides the Methods, please delete it from any other section. | Done. We retained a single location of our ethics statement. |
| 3. Please amend your manuscript to include a reference list. References must be placed at the end of the manuscript and numbered in the order that they appear in the text. For more information on the formatting of references, please visit the author guidelines at: <http://journals.plos.org/plosone/s/submission-guidelines#loc-reference-style> | We converted the reference style to Vancouver style. |

Sincerely,

Corresponding Author
