# Supplementary material for: Gender as a moderator of the association between academic self-efficacy and academic performance among nursing students in Saudi Arabia
Source: PLoS One. 2026 Sep 25;21(9):e0359418. doi: 10.1371/journal.pone.0359418 (PMC13614655; doi:10.1371/journal.pone.0359418)
Supplement: Peer review history

# Peer review: Decision Letter 0

**DOI:** 10.1371/journal.pone.0359418.r001

**Authors:** Ayse Hilal Bati

13 Aug 2026

Dear Dr.Jordan Llego,

Thank you for submitting your manuscript to PLOS ONE. After careful consideration, we feel that it has merit but does not fully meet PLOS ONE’s publication criteria as it currently stands. Therefore, we invite you to submit a revised version of the manuscript that addresses the points raised during the review process.

Please submit your revised manuscript by Oct 12 2026 11:59PM. If you will need more time than this to complete your revisions, please reply to this message or contact the journal office at plosone@plos.org. When you're ready to submit your revision, log on to https://www.editorialmanager.com/pone/ and select the 'Submissions Needing Revision' folder to locate your manuscript file.

- A letter that responds to each point raised by the academic editor and reviewer(s). You should upload this letter as a separate file labeled 'Response to Reviewers'.
- A marked-up copy of your manuscript that highlights changes made to the original version. You should upload this as a separate file labeled 'Revised Manuscript with Track Changes'.
- An unmarked version of your revised paper without tracked changes. You should upload this as a separate file labeled 'Manuscript'.

If you would like to make changes to your financial disclosure, please include your updated statement in your cover letter. Guidelines for resubmitting your figure files are available below the reviewer comments at the end of this letter.

If applicable, we recommend that you deposit your laboratory protocols in protocols.io to enhance the reproducibility of your results. Protocols.io assigns your protocol its own identifier (DOI) so that it can be cited independently in the future. For instructions see: https://journals.plos.org/plosone/s/submission-guidelines#loc-laboratory-protocols. Additionally, PLOS ONE offers an option for publishing peer-reviewed Lab Protocol articles, which describe protocols hosted on protocols.io. Read more information on sharing protocols at https://plos.org/protocols?utm_medium=editorial-email&utm_source=authorletters&utm_campaign=protocols.

As the corresponding author, your ORCID iD is verified in the submission system and will appear in the published article. PLOS supports the use of ORCID, and we encourage all coauthors to register for an ORCID iD and use it as well. Please encourage your coauthors to verify their ORCID iD within the submission system before final acceptance, as unverified ORCID iDs will not appear in the published article. *Only*  the individual author can complete the verification step; PLOS staff *cannot*  verify ORCID iDs on behalf of authors.

We look forward to receiving your revised manuscript.

Kind regards,

Ayse Hilal Bati, Professor

Academic Editor

PLOS One

**Journal Requirements:**

When submitting your revision, we need you to address these additional requirements.

1. Please ensure that your manuscript meets PLOS ONE's style requirements, including those for file naming. The PLOS ONE style templates can be found at

https://journals.plos.org/plosone/s/file?id=wjVg/PLOSOne_formatting_sample_main_body.pdf and

https://journals.plos.org/plosone/s/file?id=ba62/PLOSOne_formatting_sample_title_authors_affiliations.pdf

2. In the online submission form you indicate that your data is not available for proprietary reasons and have provided a contact point for accessing this data. Please note that your current contact point is a co-author on this manuscript. According to our Data Policy, the contact point must not be an author on the manuscript and must be an institutional contact, ideally not an individual. Please revise your data statement to a non-author institutional point of contact, such as a data access or ethics committee, and send this to us via return email. Please also include contact information for the third party organization, and please include the full citation of where the data can be found.

3. PLOS requires an ORCID iD for the corresponding author in Editorial Manager on papers submitted after December 6th, 2016. Please ensure that you have an ORCID iD and that it is validated in Editorial Manager. To do this, go to ‘Update my Information’ (in the upper left-hand corner of the main menu), and click on the Fetch/Validate link next to the ORCID field. This will take you to the ORCID site and allow you to create a new iD or authenticate a pre-existing iD in Editorial Manager.

4. Your ethics statement should only appear in the Methods section of your manuscript. If your ethics statement is written in any section besides the Methods, please delete it from any other section.

5. Please include captions for your Supporting Information files at the end of your manuscript, and update any in-text citations to match accordingly. Please see our Supporting Information guidelines for more information: http://journals.plos.org/plosone/s/supporting-information.

6. If the reviewer comments include a recommendation to cite specific previously published works, please review and evaluate these publications to determine whether they are relevant and should be cited. There is no requirement to cite these works unless the editor has indicated otherwise.

**Additional Editor Comments:**

Dear Authors,

395

Your article, "Gender-Based Moderation of the Relationship Between Academic Self-Efficacy and Academic Performance Among Nursing Students in Saudi Arabia," is a valuable piece of research, particularly in the context of nursing education. However, as the reviewers have pointed out, it requires some revision. I believe a reassessment after revision would be appropriate.

[Note: HTML markup is below. Please do not edit.]

Reviewers' comments:

Reviewer's Responses to Questions

**Comments to the Author**

1. Is the manuscript technically sound, and do the data support the conclusions?

Reviewer #1: Yes

Reviewer #2: Yes

**********

2. Has the statistical analysis been performed appropriately and rigorously?

Reviewer #1: Yes

Reviewer #2: Yes

**********

3. Have the authors made all data underlying the findings in their manuscript fully available?

The PLOS Data policy

Reviewer #1: Yes

Reviewer #2: Yes

**********

4. Is the manuscript presented in an intelligible fashion and written in standard English?

Reviewer #1: Yes

Reviewer #2: Yes

**********

Reviewer #1: Thank you for the opportunity to review this paper. The paper has several methodological and contextual strengths. However, I would like to point few concerns for improvement.

- The academic self-efficacy was measured in Year 4, but cumulative GPA includes Years 1–3. Year 4 self-efficacy cannot logically predict or explain past GPA. Please clarify this.

- Please mention of Levene’s test (for t-tests), linearity/homoscedasticity checks, or VIF values for the regression models.

- The gender is coded 0/1, which erases non-binary identities. The limitation mentions this briefly but does not explain why the institutional data only permitted binary coding.

- Please take not that self-efficacy M = 5.33 on a 1–7 scale, with most students categorized as "High." Range restriction artificially attenuates correlation magnitude.

Reviewer #2: Dear Authors and Editor,

I am grateful for this invite to review this paper titled “ Gender as a Moderator of the Association Between Academic Self-Efficacy and Academic Performance Among Nursing Students in Saudi Arabia”. This paper is of great interest especially in the context of nursing education. That said, I would like to suggest for the improvement of this paper before publication. Please see below:

1. The introduction is too long. Please make sure that only the elements of the introduction must be present. Also, nine research questions are listed; several (RQ2–RQ5) are purely descriptive and redundant with the profiling questions.i suggest that you have to condense into 4 or 5 focused questions. Note that descriptive profiling does not require separate research questions.

2. Please revisit the ethics statement as it says "All participants provided informed consent." On the part of the paper, it says, “Implied informed consent was obtained." Use one consistent term throughout. If it was truly "implied" via survey completion, state that in the Ethics Statement too.

3. While it is acceptable, I would still ask the authors to please make justification on the Self-efficacy categorization cutoffs (Low 1.0–2.9, Moderate 3.0–5.0, High 5.1–7.0) are uneven and the Moderate band is 2.0 points wide while High is 1.9.

4. Please add Cohen's d for gender GPA difference and cohort GPA difference as there is no effect sizes (Cohen's d) reported for significant t-tests.

5. There are citation problem that authors need to consider. For instance, page 16 "This gender distribution is consistent with global trends in nursing education (Esht et al., 2023), but contrasts with the more balanced gender ratio observed by Alsadaan et al. (2021)..." Verify that Alsadaan et al. (2021) actually reports gender ratios of nursing students. The title is "Challenges facing the nursing profession in Saudi Arabia: An integrative review" …if it does not report student gender distributions, replace the citation with a primary source that does.

6. The table reported as ΔR² = .004; F change = 1.641. Would this numbers be true at the same time? Please check.

7. There is a need also to report the Cohen’s d. Please add the Cohen's d values as two new columns in Table 3.

Thank you

**********

what does this mean?). If published, this will include your full peer review and any attached files.

If you choose “no”, your identity will remain anonymous but your review may still be made public.

**Do you want your identity to be public for this peer review?**  For information about this choice, including consent withdrawal, please see our Privacy Policy

Reviewer #1: No

Reviewer #2: No

**********

[NOTE: If reviewer comments were submitted as an attachment file, they will be attached to this email and accessible via the submission site. Please log into your account, locate the manuscript record, and check for the action link "View Attachments". If this link does not appear, there are no attachment files.]

To ensure your figures meet our technical requirements, please review our figure guidelines: https://journals.plos.org/plosone/s/figures

You may also use PLOS’s free figure tool, NAAS, to help you prepare publication quality figures: https://journals.plos.org/plosone/s/figures#loc-tools-for-figure-preparation.

NAAS will assess whether your figures meet our technical requirements by comparing each figure against our figure specifications.

Attachment Submitted filename: Dear Editor and Author1.docx

Attachment Submitted filename: Pone21.docx

# Author comment: Author response to Decision Letter 1

**DOI:** 10.1371/journal.pone.0359418.r002

17 Aug 2026

We have attached a separate file for our responses. We are thankful to the reviewers for their comments, which strengthen our manuscript.

Attachment Submitted filename: Response letter PONE 8-15.docx

# Peer review: Decision Letter 1

**DOI:** 10.1371/journal.pone.0359418.r003

**Authors:** Ayse Hilal Bati

14 Sep 2026

Gender as a Moderator of the Association Between Academic Self-Efficacy and Academic Performance Among Nursing Students in Saudi Arabia

PONE-D-26-29120R1

Dear Dr. Jordan Llego,

We’re pleased to inform you that your manuscript has been judged scientifically suitable for publication and will be formally accepted for publication once it meets all outstanding technical requirements.

Within one week, you’ll receive an e-mail detailing the required amendments. When these have been addressed, you’ll receive a formal acceptance letter and your manuscript will be scheduled for publication.

An invoice will be generated when your article is formally accepted. Please note, if your institution has a publishing partnership with PLOS and your article meets the relevant criteria, all or part of your publication costs will be covered. Please make sure your user information is up-to-date by logging into Editorial Manager at Editorial Manager® and clicking the ‘Update My Information' link at the top of the page. For questions related to billing, please contact billing support.

If your institution or institutions have a press office, please notify them about your upcoming paper to help maximize its impact. If they’ll be preparing press materials, please inform our press team as soon as possible -- no later than 48 hours after receiving the formal acceptance. Your manuscript will remain under strict press embargo until 2 pm Eastern Time on the date of publication. For more information, please contact onepress@plos.org.

Kind regards,

Ayse Hilal Bati, Professor

Academic Editor

PLOS One

Additional Editor Comments (optional):

Dear Jordan Llego,

Your article has been evaluated as suitable for publication in PLOS ONE.

Thank you for your contributions.

Reviewers' comments:

Reviewer's Responses to Questions

**Comments to the Author**

Reviewer #1: All comments have been addressed

Reviewer #2: All comments have been addressed

**********

2. Is the manuscript technically sound, and do the data support the conclusions?

Reviewer #1: Yes

Reviewer #2: Yes

**********

3. Has the statistical analysis been performed appropriately and rigorously?

Reviewer #1: (No Response)

Reviewer #2: Yes

**********

4. Have the authors made all data underlying the findings in their manuscript fully available?

The PLOS Data policy

Reviewer #1: Yes

Reviewer #2: Yes

**********

5. Is the manuscript presented in an intelligible fashion and written in standard English?

Reviewer #1: Yes

Reviewer #2: Yes

**********

Reviewer #1: Thank you once again for the time to re review this paper. I empathically believe that all my comments have been scientifically addressed. Congratulations.

Reviewer #2: I have no further comments to the authors. I would like to thank the authors for considering my suggestions.

**********

what does this mean?). If published, this will include your full peer review and any attached files.

If you choose “no”, your identity will remain anonymous but your review may still be made public.

**Do you want your identity to be public for this peer review?**  For information about this choice, including consent withdrawal, please see our Privacy Policy

Reviewer #1: No

Reviewer #2: No

**********

# Editor report: Acceptance letter

**DOI:** 10.1371/journal.pone.0359418.r004

**Authors:** Ayse Hilal Bati

PONE-D-26-29120R1

PLOS One

Dear Dr. Llego,

I'm pleased to inform you that your manuscript has been deemed suitable for publication in PLOS One. Congratulations! Your manuscript is now being handed over to our production team.

At this stage, our production department will prepare your paper for publication. This includes ensuring the following:

* All references, tables, and figures are properly cited

* All relevant supporting information is included in the manuscript submission,

* There are no issues that prevent the paper from being properly typeset

You will receive further instructions from the production team, including instructions on how to review your proof when it is ready. Please keep in mind that we are working through a large volume of accepted articles, so please give us a few days to review your paper and let you know the next and final steps.

Lastly, if your institution or institutions have a press office, please let them know about your upcoming paper now to help maximize its impact. If they'll be preparing press materials, please inform our press team within the next 48 hours. Your manuscript will remain under strict press embargo until 2 pm Eastern Time on the date of publication. For more information, please contact onepress@plos.org.

You will receive an invoice from PLOS for your publication fee after your manuscript has reached the completed accept phase. If you receive an email requesting payment before acceptance or for any other service, this may be a phishing scheme. Learn how to identify phishing emails and protect your accounts at https://explore.plos.org/phishing.

If we can help with anything else, please email us at customercare@plos.org.

Thank you for submitting your work to PLOS One and supporting open access.

Kind regards,

PLOS One Editorial Office Staff

on behalf of

Dr. Ayse Hilal Bati

Academic Editor

PLOS One
